# Air Space

**Published:** 1918-10

**Authors:** 


### Air Space.

While the long established rule of 1000 cubic feet of air space per capita, approximately equal in its three dimensions, still holds good, we have come to realize rather recently, that air allotment involves a factor quite independent of and perhaps much more important than a provision for oxygen and the dissipation of carbon dioxid and other more toxic but practically unknown emanations. This factor is the necessity of air space to allow dilution of infectious organisms and thus diminish the danger of conveyance of disease. By the law of chance, one man in a room of the size mentioned is considerably safer than two in a room twice as large, or of 30 in a room 30 times as large. The general principle applies to persons in health—meaning, when any considerable number are gathered together, that some will be carriers of germs to which they have not succumbed—and even more strongly to collections of persons some of whom have more or less contagious diseases, whether of the same or, a fortiori, of different kinds. Screens, sheets or even newspapers hung between beds or dormitories or hospital wards obviously increase the factor of safety, without actually increasing air space but. up to the point at which ventilation and opportunities for sedimentation from the air and dilution of virus become practically as great as out doors, segregation by as small units as practicable, is more important than increase of air space. Even being out doors is not necessarily a guarantee of freedom from danger.

This is a point which has required not so much experiment and experience as realization. It is of both civil and military importance.

